# PSAT1 is upregulated by METTL3 to attenuate high glucose-induced retinal pigment epithelial cell apoptosis and oxidative stress

**DOI:** 10.1186/s13000-024-01556-4

**Published:** 2024-10-15

**Authors:** Xiaofeng Du, Yanting Wang, Fan Gao

**Affiliations:** 1 Department of Ophthalmology, Henan Provincial Eye Hospital, Henan Provincial People’s Hospital, Zhengzhou City, Henan 450003 China; 2 Department of Ophthalmology, Yan’an People’s Hospital, No. 16 Qilipu Street, Baota District, Yan’an City, Shaanxi province 716000 China

**Keywords:** Methyltransferase-like 3, Phosphoserine aminotransferase 1, High-glucose, Diabetic retinopathy, ARPE-19 cells

## Abstract

**Background:**

Diabetic retinopathy (DR) is a major ocular complication of diabetes mellitus, and a significant cause of visual impairment and blindness in adults. Phosphoserine aminotransferase 1 (PSAT1) is an enzyme participating in serine synthesis, which might improve insulin signaling and insulin sensitivity. Furthermore, it has been reported that the m6A methylation in mRNA controls gene expression under many physiological and pathological conditions. Nevertheless, the influences of m6A methylation on PSAT1 expression and DR progression at the molecular level have not been reported.

**Methods:**

High-glucose (HG) was used to treat human retinal pigment epithelial cells (ARPE-19) to construct a cell injury model. PSAT1 and Methyltransferase-like 3 (METTL3) levels were detected by real-time quantitative polymerase chain reaction (RT-qPCR). PSAT1, B-cell lymphoma-2 (Bcl-2), Bcl-2 related X protein (Bax), and METTL3 protein levels were examined by western blot assay. Cell viability and apoptosis were detected by Cell Counting Kit-8 (CCK-8) and TUNEL assays. Reactive oxygen species (ROS), malondialdehyde (MDA), and Glutathione peroxidase (GSH-Px) levels were examined using special assay kits. Interaction between METTL3 and PSAT1 was verified using methylated RNA immunoprecipitation (MeRIP) and dual-luciferase reporter assay.

**Results:**

PSAT1 and METTL3 levels were decreased in DR patients and HG-treated ARPE-19 cells. Upregulation of PSAT1 might attenuate HG-induced cell viability inhibition and apoptosis and oxidative stress promotion in ARPE-19 cells. Moreover, PSAT1 was identified as a downstream target of METTL3-mediated m6A modification. METTL3 might improve the stability of PSAT1 mRNA via m6A methylation.

**Conclusion:**

METTL3 might mitigate HG-induced ARPE-19 cell damage partly by regulating the stability of PSAT1 mRNA, providing a promising therapeutic target for DR.

**Supplementary Information:**

The online version contains supplementary material available at 10.1186/s13000-024-01556-4.

## Introduction

As a chronic metabolic disorder characterized by sustained hyperglycemia, diabetes mellitus is traditionally classified as macro and microvascular complications depending on the underlying pathophysiology [5]. Among them, diabetic retinopathy (DR) is the most frequent microvascular complication of diabetes mellitus, and is the primary cause of acquired blindness and visual impairment, especially in the working-age adult population Tan et al. 28; Teo et al. [29]. Clinically, non-proliferative and proliferative have been considered the initial and advanced stages of DR [22]. With prolonged retinal ischemia and the release of vasoactive substances, non-proliferative DR develops into proliferative DR, which is characterized by the formation of new blood vessels on the retina or the optic disk surface. These vessels are leaky and fragile and cause poor retinal blood flow, which progress to vitreous haemorrhages, eventually leading to retinal detachment and retinal detachment [4, 19]. Accounting for approximately one-third of people diagnosed with diabetes mellitus, DR dramatically reduces patients’ quality of life and generates a massive economic burden [16] Tóth et al. [27]. Thus, exploring the molecular mechanisms underlying DR is crucial for developing effective pharmaceutical preparations.

There is an accumulating body of evidence indicating that hyperglycaemia is the major instigator of the development of DR [6, 15]. Currently, the exact mechanisms by which hyperglycemia produces diabetes complications, containing DR are fully understood. However, high blood glucose levels are well known to have metabolic effects mediating retina microvascular injury [23]. Hyperglycemia-induced alterations in biochemical pathways might contribute to impaired pericyte dysfunction and retinal vascular complications, such as blood-retinal barrier and pathological neovascularization, by generating oxidative stress and inducing inflammatory intermediate [1, 21]. In addition, oxidative stress might produce a pro-inflammatory state that increases the synthesis of many chemokines and cytokines, which promote DR development via stimulating the growth of pre-retinal proliferative tissue [8]. Herein, bioinformatics methods identified that Phosphoserine aminotransferase 1 (PSAT1) expression was clearly downregulated in HG-human retinal pigment epithelial (ARPE-19 cells) through detecting microarray data in public databases. As an enzyme involved in serine biosynthesis, PSAT1 has some vital metabolic functions, including the regulation of blood glucose [40]. Furthermore, α-Ketoglutaric acid (α-KG), a byproduct of the PSAT1-catalyzed reaction, has been reported to stimulate insulin secretion [20]. Beyond that, insulin sensitivity is an important aspect of maintaining glucose homeostasis [18]. Indeed, the upregulation of PSAT1 has been confirmed to enhance insulin signaling and insulin sensitivity in vitro and in vivo under normal conditions [36]. Yet, the role and underlying mechanism of PSAT1 in DR are not well understood.

In recent years, increasing studies have pointed toward that N6-methyladenosine (m6A) RNA modifications represent important regulators of gene expression and pathogenesis Murakami et al. [17] Y. Yang et al. [34]. The sixth N methylation of adenosine in RNA, m6A is the most abundant transcription modification in eukaryotic messenger RNA and controls RNA translocation, translation, and stability [31]. As with other methylation events, m6A is dynamically and reversibly regulated by three groups of m6A-binding proteins [24]: the ‘writer complex’ that installs m6A marks, including methyltransferase-like 14 (METTL14), METTL3, and Wilms tumor 1-associated protein (WTAP); the ‘erasers’ that remove m6A marks, containing obesity-associated protein (FTO) and alkylation repair homolog protein 5 (ALKBH5); and ‘readers’ that recognize and bind to m6A modified transcripts. Recent studies have shown the influences of m6A RNA modification on many disease conditions, containing DR [13]. As a major RNA m6A methyltransferase, METTL3 has been reported to install the m6A modification and improve the stability of SNHG7, thereby repressing endothelial-mesenchymal transition in DR [3]. Furthermore, it has been reported that the upregulation of METTL3 might mitigate HG-triggered retinal pigment epithelium cell pyroptosis and apoptosis via regulating the Akt signaling cascade [37]. Herein, a public prediction server SRAMP presented that PSAT1 has the potential m6A sites. Moreover, PSAT1 was identified to be a possible target gene of METTL3. Therefore, we aimed to explore whether METTL3-guided m6A marked PSAT1 might be involved in the modulation of HG-induced ARPE-19 cell injury during DR progress.

## Materials and methods

### Clinical samples and cell culture

In this research, 28 DR patients and 28 healthy volunteers (normal controls) were recruited at the Henan Provincial Eye Hospital, Henan Provincial People’s Hospital. Then, peripheral blood samples were collected from all subjects, and serum specimens were obtained by centrifugation. Every participant signed the written informed consent prior to enrolling in this study in accordance with the Declaration of Helsinki, which was authorized by the Ethics Committee of Henan Provincial Eye Hospital, Henan Provincial People’s Hospital.

Under an atmosphere of 37˚C containing 5% CO_2_, human retinal pigment epithelial cells (ARPE-19 cells, CL-0026, Procell, Wuhan, China) were cultured in DMEM/F12 medium plus 10% FBS and 1% P/S (CM-0026, Procell). For treatment, ARPE-19 cells at 75% confluency were first incubated in FBS-free DMEM/F12 medium for 24 h, which then switched to high-glucose (HG, 50 mM D-glucose, Sigma-Aldrich, St. Louis, MO, USA), normal glucose (NG, 5.5 mM D-glucose), or mannitol (MA) conditions for various time points (0, 24, 48 h, and 72 h).

### Real-time quantitative polymerase chain reaction (RT-qPCR)

In this experiment, total RNAs from clinical samples and ARPE-19 cells were extracted according to the Trizol reagent (Invitrogen, Paisley Scotland, UK). After qualification with the NanoDrop 2000 system, total RNAs were reversely transcribed into cDNA using GoScript™ Reverse Transcription System (Promega, Madison, WI, USA). On the ABI 7500 fast PCR System (Applied Biosystems, Foster City, CA, USA), amplification reaction was carried out based on SYBR Green PCR Kit (Takara). After normalization to GAPDH, the results from each group were subjected to the 2^–ΔΔCt^ method analysis. Primers used are shown in Table 1.

**Table 1:** Primers sequences used for PCR

Name		Primers for PCR (5’-3’)
METTL3	Forward	TTGTCTCCAACCTTCCGTAGT
	Reverse	CCAGATCAGAGAGGTGGTGTAG
PSAT1	Forward	AAAAACAATGGAGGTGCCGC
	Reverse	GGCTCCACTGGACAAACGTA
GAPDH	Forward	GGAGCGAGATCCCTCCAAAAT
	Reverse	GGCTGTTGTCATACTTCTCATGG

### Western blot assay

In brief, lysis of ARPE-19 cells was conducted in line with RIPA Lysis buffer (Keygen, Nanjing, China). After being clarified using centrifugation, the protein concentrations were determined with a BCA protein assay kit. Subsequently, 50 µg of each protein sample was loaded and separated on 10% SDS-PAGE gels, followed by transfer to PVDF membranes (Invitrogen). After blocking, the membranes were labeled at 4˚C with primary antibodies: PSAT1 (10501-1-AP, 1:5000; Proteintech, Wuhan, China), METTL3 (ab195352, 1:1000; Abcam, Cambridge, MA, USA), B-cell lymphoma-2 (Bcl-2, ab32124, 1:1000; Abcam), Bcl-2 related X protein (Bax, ab32503, 1:1000; Abcam), and GAPDH (60004-1-lg, 1:50000; Proteintech). The next day, protein bands were analyzed using ECL reagent (Solarbio) and Image J software after 2 h of incubation with secondary antibody.

### Cell transfection

For PSAT1 or METTL3 overexpression system (OE-PSAT1 or OE-METTL3), PSAT1 cDNA (NM_058179.4) or METTL3 cDNA (NM_019852.5) were inserted into pCDH puro lentiviral vector (System Biosciences, Mountain View, CA, USA), respectively. pCDH puro lentiviral empty vector was used as control (Vector). For PSAT1 or METTL3 knockdown system, the short hairpin RNAs (shRNA) sequence of PSAT1 or METTL3 was subcloned into pLKO.1 lentiviral vector (Addgene, Cambridge, Massachusetts, USA) to acquire sh-PSAT1 or sh-METTL3 lentivirus plasmid. pLKO.1 empty vector was applied as sh-NC. Subsequently, these obtained lentivirus plasmids were transfected into HEK293T cells (CL-0005, Procell) with lentivirus package plasmid mixtures. After being collected, cell supernatants were inferred into ARPE-19 cells along with 8 µg/mL polybrene. Finally, 5 µg/mL puromycin was added to select stably expressing cell lines.

### Counting kit (CCK-8) assay

In short, ARPE-19 cell viability was evaluated according to the CCK-8 kit (KeyGEN). Generally, after treatment with HG or NG at 37˚C for 48 h, transfected cells were harvested and cultured in 96-well plates at the density of 2 × 10^3^ cells per well. At different time points, each well was supplemented with CCK-8 solution for at least 4 h. Finally, the absorbance at 450 nm was estimated based on a microplate reader.

### ROS detection

2’,7’-dichlorofluorescin diacetate (DCFH-DA) (Sigma-Aldrich) was utilized to measure ROS level in this assay. After being harvested and rinsed using PBS, ARPE-19 cells were added with DCFH-DA for 30 min away from light. At last, the fluorescence intensity was detected using flow cytometry and analyzed using Flowjo_V10.

### Measurement of MDA and GSH-Px

Concisely, ARPE-19 cells were harvested and lysed after treatment and transfection. In cell extract, MDA content and GSH-Px activity were assessed using their corresponding commercial kits (S0131S, Beyotime; A005-1-2, Jiancheng Biotech, Nanjing, China).

### TUNEL assay

Treated ARPE-19 cell apoptosis was analyzed using a TUNEL fluorescence kit (Beyotime, Shanghai, China). Generally, 5 × 10^4^ ARPE-19 cells in 6-well plates were stained with 5 µM TUNEL for 2 h in the dark. Cell nuclei were stained with DAPI (Beyotime) for 5 min. The final count was displayed as the percentage of total cells measured through visualization under a fluorescent microscope (Leica, Japan). Green fluorescence presented TUNEL-positive cells, and blue fluorescence presented the nucleus.

### Methylated RNA immunoprecipitation (MeRIP)

This experiment was carried out with a Magna MeRIP m6A kit (Millipore, Molsheim, France). Briefly, ARPE-19 cells were transfected with sh-NC, sh-METTL3, Vector, or OE-METTL3, followed by the extraction of total RNAs using TRIzol (Invitrogen). Then, RNA fragmentation buffer was applied to fragment RNA to approximately 300 bp, and one-tenth volume of fragmented RNA was used as “Input”. Meanwhile, anti-m6A (MABE1006, Millipore) or anti-IgG (Millipore) antibody was bound to the magnetic beads at 4˚C overnight, which were incubated with the rest fragmented RNA. After being eluted and purified, targeted RNA was analyzed using RT-qPCR assay and normalized to the input.

### Dual-luciferase reporter assay

First of all, an online bioinformatics software SRAMP predicted the m6A sites of PSAT1 mRNA and found enriched m6A peaks along the PSAT1 sequence, which was sub-cloned into pmirGLO vector (Promega, Madison, WI, USA), termed wild-type (WT)-PSAT1 constructs. In parallel, m6A methylated site mutations of PSAT1 were synthesized by Genscript (Nanjing, China) and inserted into pmirGLO plasmid, generating mutant (MUT)-PSAT1 constructs. Subsequently, these constructs were respectively transfected into ARPE-19 cells along with sh-NC, sh-METTL3, Vector, or OE-METTL3, followed by the analysis of the luciferase activity in cell lysates based on dual-luciferase reporter assay kit (Promega).

### actinomycin D treatment

In brief, different groups of ARPE-19 cells in 6-well plates were harvested at 0, 2, 4, and 8 h after treatment with actinomycin D (2 mg/mL, Sigma-Aldrich). After being extracted, RT-qPCR assay was performed to analyze the stability of PSAT1 mRNA.

### statistical analysis

In this research, the data were displayed as the mean ± standard deviation (SD). Pearson correlation analysis was used for expression association. The data were analyzed using GraphPad Prism7 with Student’s *t*-test for two groups and one-way ANOVA with Tukey’s tests for multiple groups. Differences were considered significant at *P* < 0.05.

## Results

### Identification of PSAT1 expression in HG-induced ARPE-19 cells

**Fig. 1:**
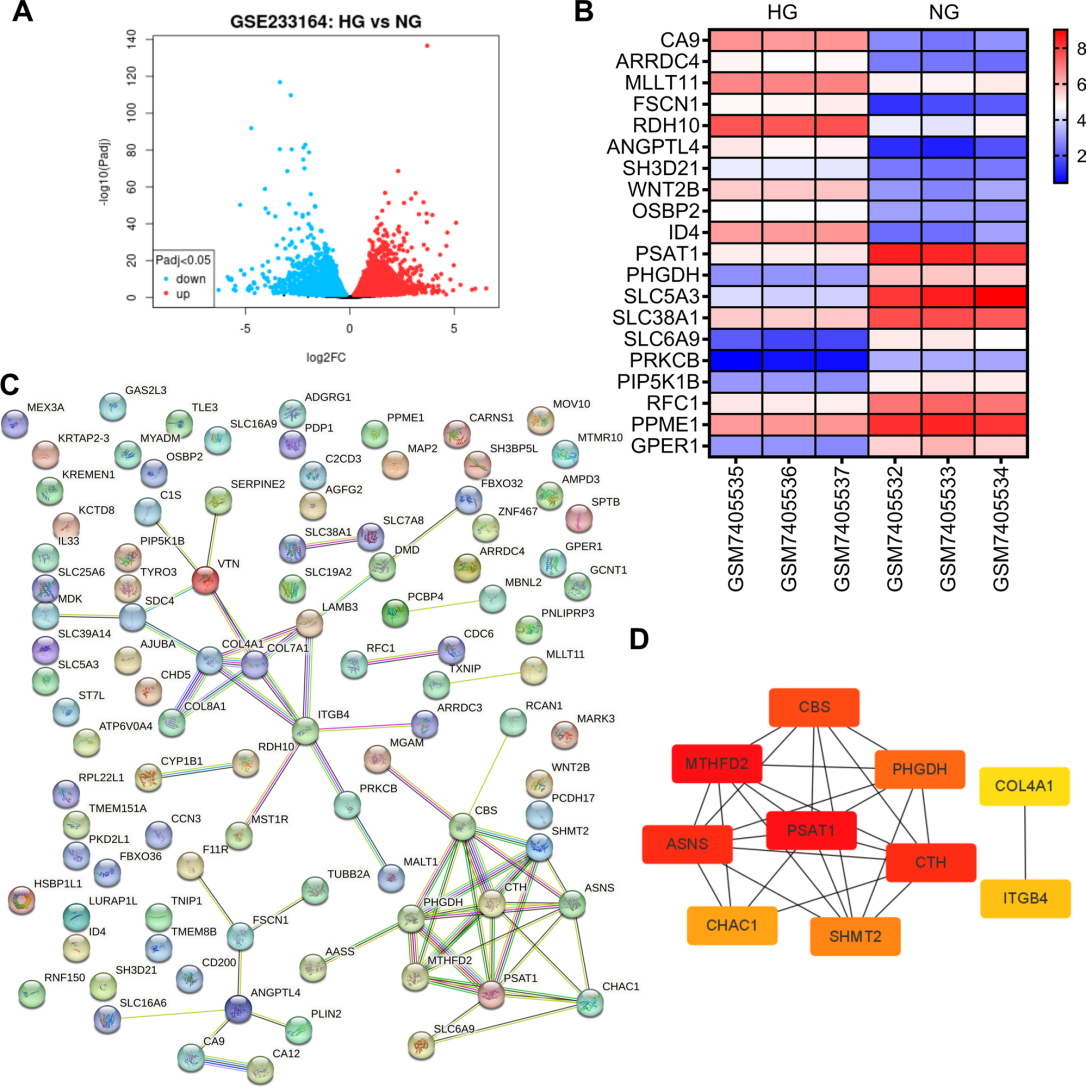
PSAT1 expression in HG-induced ARPE-19 cells. (**A**) Volcano plot shows differentially expressed genes in ARPE-19 cells induced by HG. (**B**) Heat map displaying the top 10 differentially expressed up- and down-regulated genes. (**C**) String Database Analysis of Protein-Protein Interaction Network (PPI) for top 100 Differentially Expressed Genes. (**D**) Ten candidate hub genes were identified from the PPI network using Cytoscape software utilizing the MCC algorithm of the plug-in CytoHubba

Firstly, to screen out the candidate functional genes in HG-induced ARPE-10 cells, we analyzed the overall gene expression pattern in a microarray gene-profiling data set (GSE233164) from the Gene Expression Omnibus (GEO) (Fig. 1A). For further selection, we chose the top 10 differentially expressed up- and down-regulated genes (Fig. 1B). In addition, a PPI network was established for the top 100 differentially expressed genes (DEGs) according to the String database (Fig. 1C). Subsequently, the top 10 hub genes selected in the CytoHubba plug-in using the MCC algorithm and node degree contained MTHFD2, ASNS, PSAT1, CTH, CBS, PHGDH, SHMT2, CHAC1, ITGB4, and COL4A1 (Fig. 1D). Among them, PSAT1 expression was clearly downregulated in HG-treated ARPE-10 cells. Therefore, PSAT1 was selected for this research.

### PSAT1 expression was downregulated in DR patients and HG-induced ARPE-19 cells

**Fig. 2:**
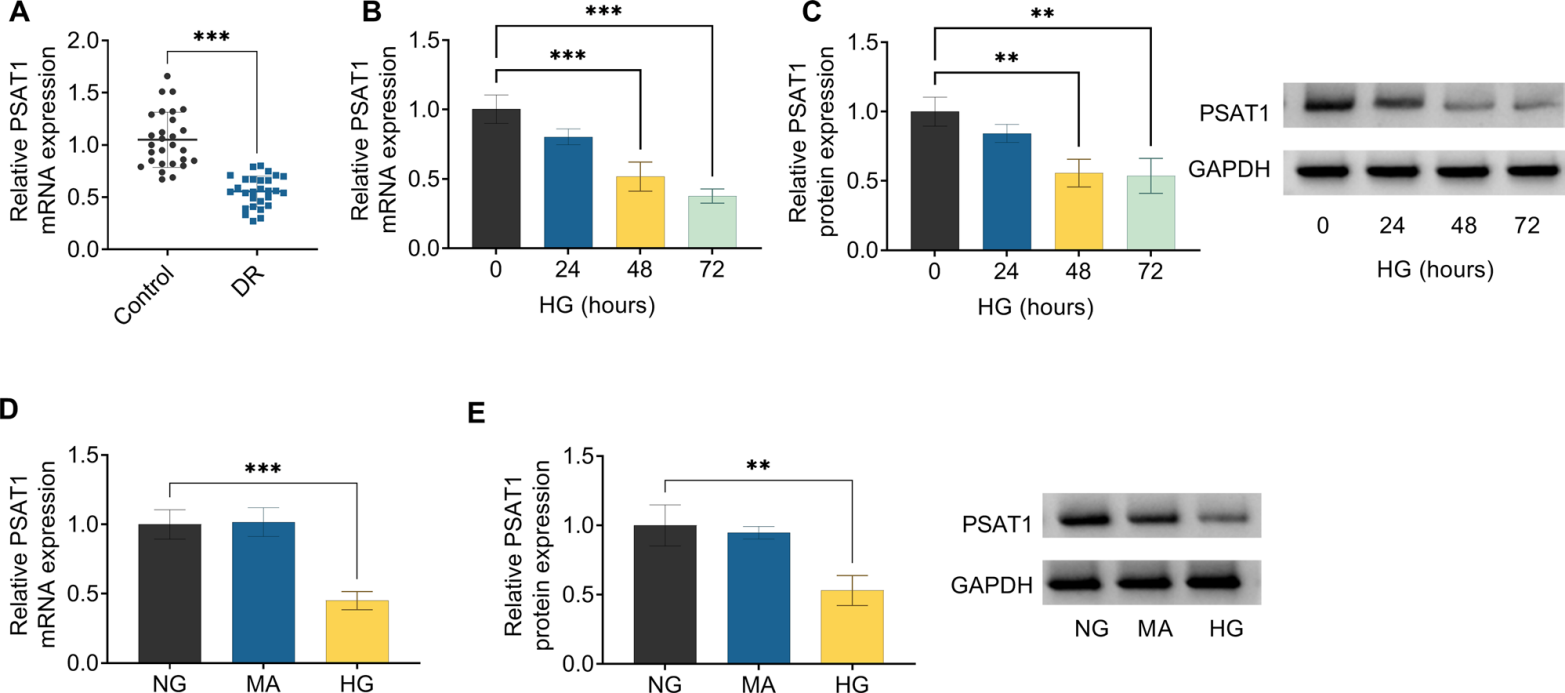
Expression patterns of PSAT1 in DR patients and HG-induced ARPE-19 cells. (**A**) PSAT1 mRNA expression was detected in the serum of 28 DR and 28 Control group using RT-qPCR assay. (**B**) RT-qPCR analysis of PSAT1 level in ARPE-19 cells treated with 50 mM HG for 0 h, 24 h, 48 h, and 72 h. (**C**) Western blot analysis of PSAT1 protein level in ARPE-19 cells treated with 50 mM HG for 0 h, 24 h, 48 h, and 72 h. (**D**) PSAT1 content was determined in ARPE-19 cells treated with NG, MA, and HG using RT-qPCR assay. (**E**) PSAT1 protein level was measured in ARPE-19 cells treated with NG, MA, and HG using Western blot. **P < 0.01, ***P < 0.001, *n* = 3

Furthermore, to check the functional role of PSAT1 on DR, its expression pattern was first analyzed using RT-qPCR assay. According to the data presented in Fig. 2A, PSAT1 mRNA expression was significantly decreased in the serum samples from patients with DR relative to the serum samples from the control group. Furthermore, the ARPE-19 cells were exposed to HG (50 mM) for 0 h, 24 h, 48 h, and 72 h based on previous research [39]. As shown in Fig. 2B and C, PSAT1 expression was time-dependently reduced in HG-induced ARPE-19 cells for 0–72 h. In particular, the treatment time was more than or equal to 48 h. Therefore, 48 h was selected for the subsequent experiment. Beyond that, we further verified that PSAT1 mRNA level and protein level were lower expressed in HG-induced ARPE-19 cells versus NG or MA-treated cells (Fig. 2D and E). Together, these data suggested that PSAT1 might participate in HG-triggered ARPE-19 cells.

### Upregulation of PSAT1 might relieve HG-induced ARPE-19 cell damage

**Fig. 3:**
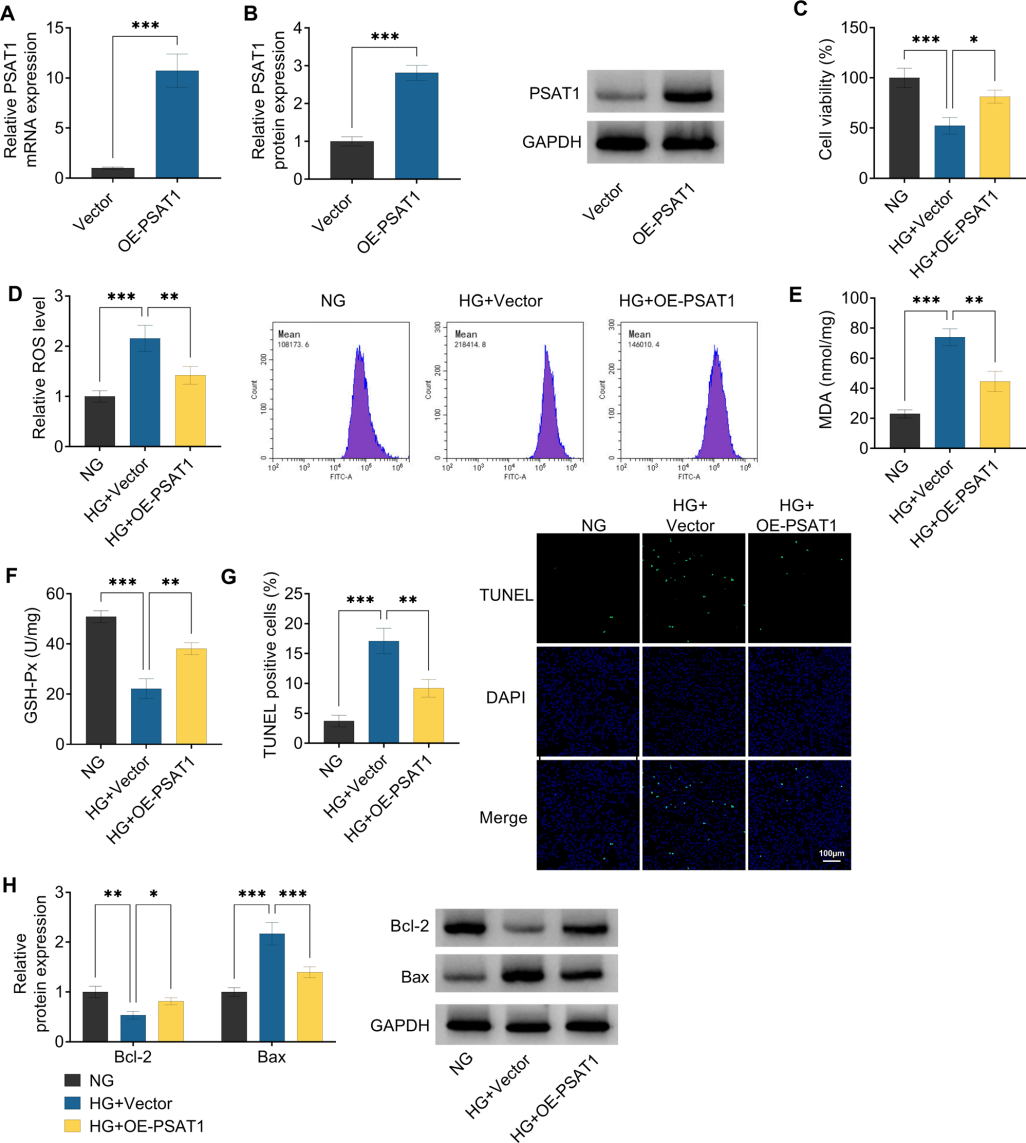
Effects of PSAT1 on HG-induced ARPE-19 cell injury. (**A** and **B**) RT-qPCR assay and Western blot assay were used to detect PSAT1 expression in ARPE-19 cells transfected with Vector or OE-PSAT1. (**C**-**H**) ARPE-19 cells were treated with NG, HG + Vector, or HG + OE-PSAT1. (**C**) Cell viability was assessed in treated ARPE-19 cells using CCK-8 assay. (D-F) ROS, MDA, and GSH-Px levels were measured using Commercial kits in treated ARPE-19 cells. (**G**) Cell apoptosis was examined using TUNEL assay in treated ARPE-19 cells. (**H**) Bcl-2 and Bax protein levels in treated ARPE-19 cells were detected using western blot. *P < 0.05, **P < 0.01, ***P < 0.001, *n* = 3

Next, in vitro gain-of-function analysis was conducted to identify the biological role of PSAT1 in HG-induced ARPE-19 cells. First of all, RT-qPCR and Western blot experiments presented that PSAT1 content was remarkably increased in OE-PSAT1-transfected ARPE-19 cells compared with the vector groups (Fig. 3A and B), indicating that the overexpression efficiency of OE-PSAT1 is available for following assay. Functionally, HG treatment might apparently reduce cell viability in ARPE-19 cells, while these effects were partly abolished by PSAT1 overexpression (Fig. 3C). Moreover, the oxidative stress status of ARPE-19 cells was further analyzed by measuring the general oxidative stress indicators (ROS, MDA, and GSH) levels. Results exhibited that HG treatment-triggered ROS and MDA level enhancement and GSH-Px level reduction in ARPE-19 cells were effectively overturned after OE-PSAT1 transfection (Fig. 3D and F). Beyond that, TUNEL assay showed that the upregulation of PSAT1 might partly abrogate the apoptotic effects caused by HG treatment in ARPE-19 cells (Fig. 3G). Similar to the TUNEL results, western blot analysis displayed that HG-mediated Bcl-2 (anti-apoptosis marker) protein level decrease and Bax (pro-apoptosis marker) elevation in ARPE-19 cells were greatly reversed after OE-PSAT1 introduction (Fig. 3H). In addition, our data confirmed that ARPE-19 cell viability, ROS, MDA, GSH-Px, and apoptosis did not differ between the HG group and the HG + Vector group (Figure S1). Overall, the above-mentioned data suggested that HG-evoked retinal pigment epithelial cell injury might be attenuated by PSAT1 upregulation.

### METTL3-mediated m6A methylation might improve the mRNA stability of PSAT1 in ARPE-19 cells

**Fig. 4:**
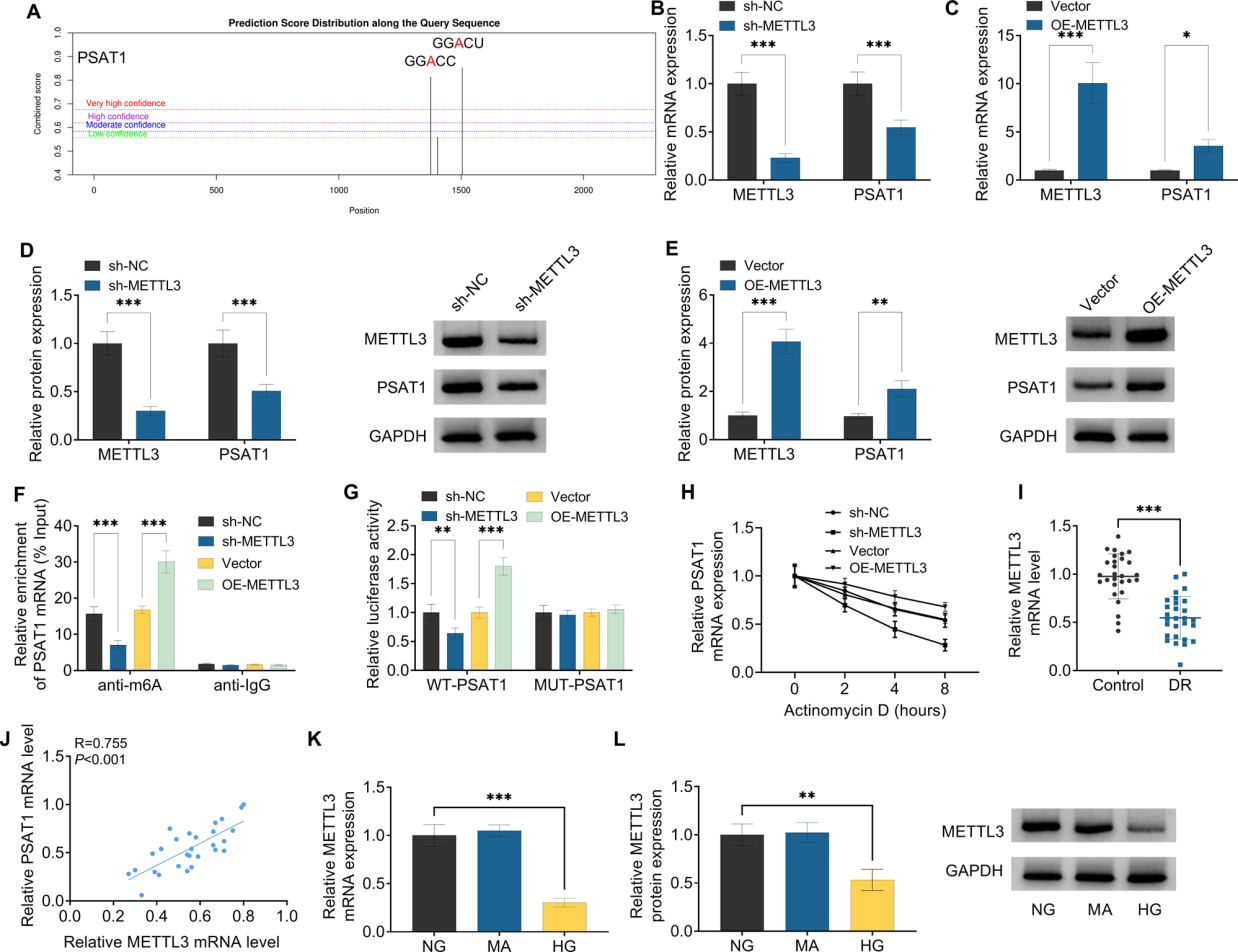
PSAT1 was modulated by METTL3-mediated m6A methylation. (**A**) SRAMP predicted that PSAT1 mRNA has an m6A site. (**B** and **C**) METTL3 and PSAT1 mRNA expression were examined using in ARPE-19 cells transfected with sh-NC, sh-METTL3, Vector, or OE-PSAT1 using RT-qPCR assay. (**D** and **E**) Western blot analysis of METTL3 and PSAT1 protein level in ARPE-19 cells transfected with sh-NC, sh-METTL3, Vector, or OE-PSAT1. (**F**) Changes in the m6A methylation level of PSAT1 after METTL3 knockdown or overexpression were assessed using MeRIP-qPCR assay. (**G**) Their binding was verified using a luciferase reporter assay in 293t cells. (**H**) Influences of METTL3 downregulation or upregulation on PSAT1 mRNA stability after Actinomycin D treatment was assessed using RT-qPCR in ARPE-19 cells. (**I**) METTL3 content was measured in the serum of 28 DR and 28 Control group using RT-qPCR assay. (**J**) Pearson correlation analysis was applied to evaluate the expression association between METTL3 and PSAT1 in DR patients. (**K** and **L**) PSAT1 mRNA level and protein level were examined in ARPE-19 cells treated with NG, MA, and HG using RT-qPCR assay and Western blot. *P < 0.05, **P < 0.01, ***P < 0.001, *n* = 3

Based on SRAMP (an online tool to predict m6A sites) database analysis, the potential m6A sites in PSAT1 mRNA were found (Fig. 4A). Subsequently, RT-qPCR and western blot results displayed that PSAT1 mRNA level (Fig. 4B and C) and protein level (Fig. 4D and E) were apparently blocked by METTL3 knockdown, and obviously improved through METTL3 upregulation in ARPE-19 cells. Furthermore, MeRIP-qPCR analysis presented that the silencing of METTL3 might distinctly decrease the m6A level of PSAT1 mRNA, whereas the upregulation of METTL3 might markedly increase the m6A level of PSAT1 mRNA, and there was no significant difference between them in the anti-IgG groups (Fig. 4F). To further validate the essential role of m6A methylation in the regulation of PSAT1, we conducted a luciferase reporter assay in 293t cells. Data displayed that the luciferase signal of METTL3 downregulation ARPE-19 cells with the wild-type reporter tended to decrease, whereas transfection with the mutant reporter exhibited no difference. On the contrary, transfecting the wild-type reporter rather than the mutant reporter into METTL3-overexpressing cells resulted in an increase in luciferase activity (Fig. 4G). Besides, the actinomycin D experiment showed that METTL3 silencing enhanced the degradation rate of PSAT1 mRNA, while upregulating METTL3 but not sh-METTL3 improved its stability (Fig. 4H). In addition, RT-qPCR assay displayed that METTL3 level was clearly downregulated in DR patients and positively associated with PSAT1 expression (Fig. 4I and J). Beyond that, we further verified that METTL3 content remarkably declined in HG-treated ARPE-19 cells compared with NG or MA-treated cells (Fig. 4K and L). Collectively, these data implied that METTL3 is able to regulate PSAT1 mRNA stability through m6A modification.

### METTL3 overexpression might repress HG-evoked ARPE-19 cell damage via targeting PSAT1 in vitro

**Fig. 5:**
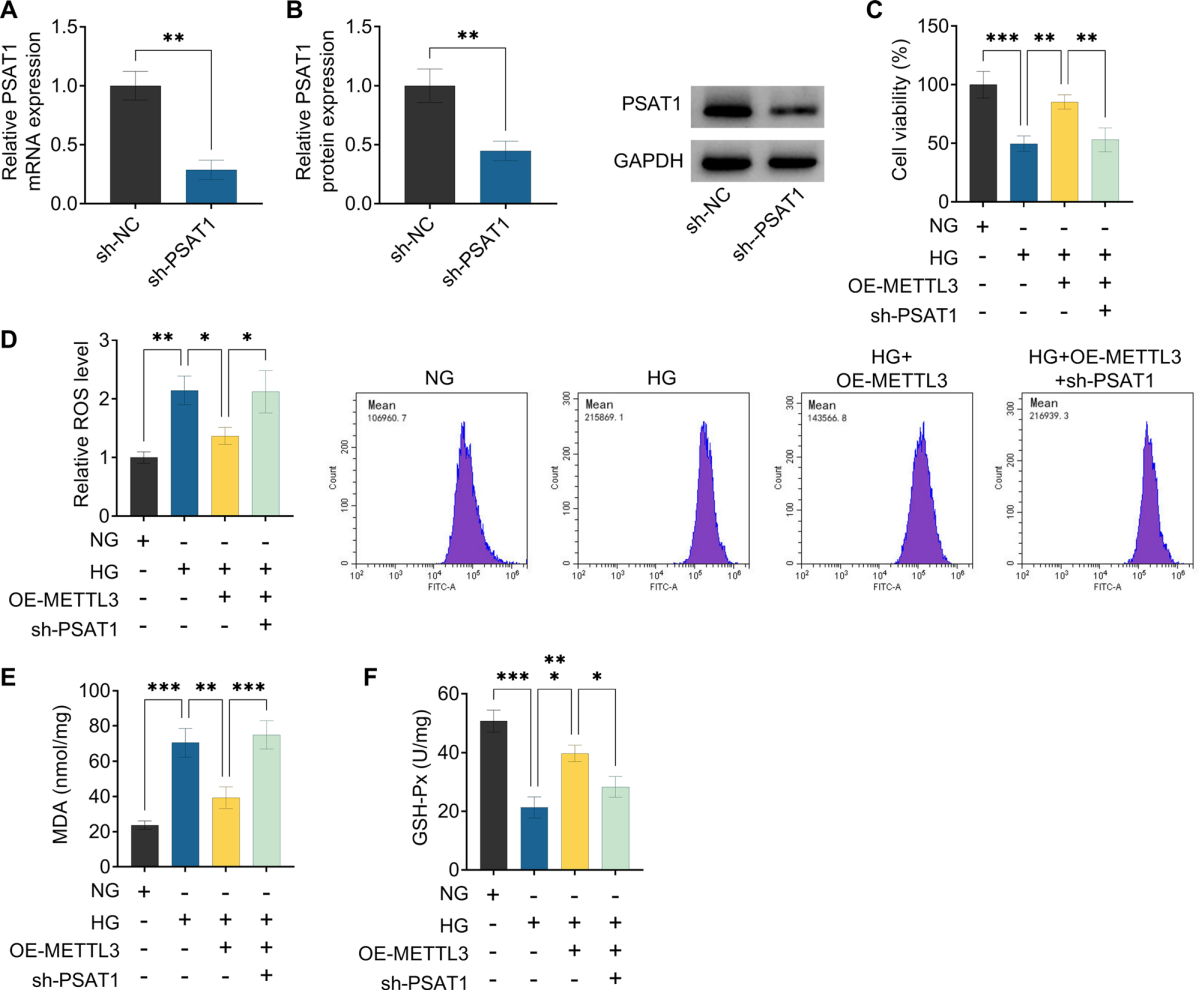
METTL3/PSAT1 regulated HG-mediated ARPE-19 cell viability and oxidative stress in vitro. (**A** and **B**) PSAT1 expression was determined in ARPE-19 cells transfected with sh-NC or sh-PSAT1 using RT-qPCR assay and Western blot assay. (**C**-**F**) ARPE-19 cells were treated with NG, HG, HG + sh-PSAT1, or HG + sh-PSAT1 + sh-PSAT1. (**C**) CCK-8 analysis of cell viability in treated ARPE-19. (**D**-**F**) Commercial kits were applied to analyze ROS, MDA, and GSH-Px levels in treated ARPE-19 cells. *P < 0.05, **P < 0.01, ***P < 0.001, *n* = 3

**Fig. 6:**
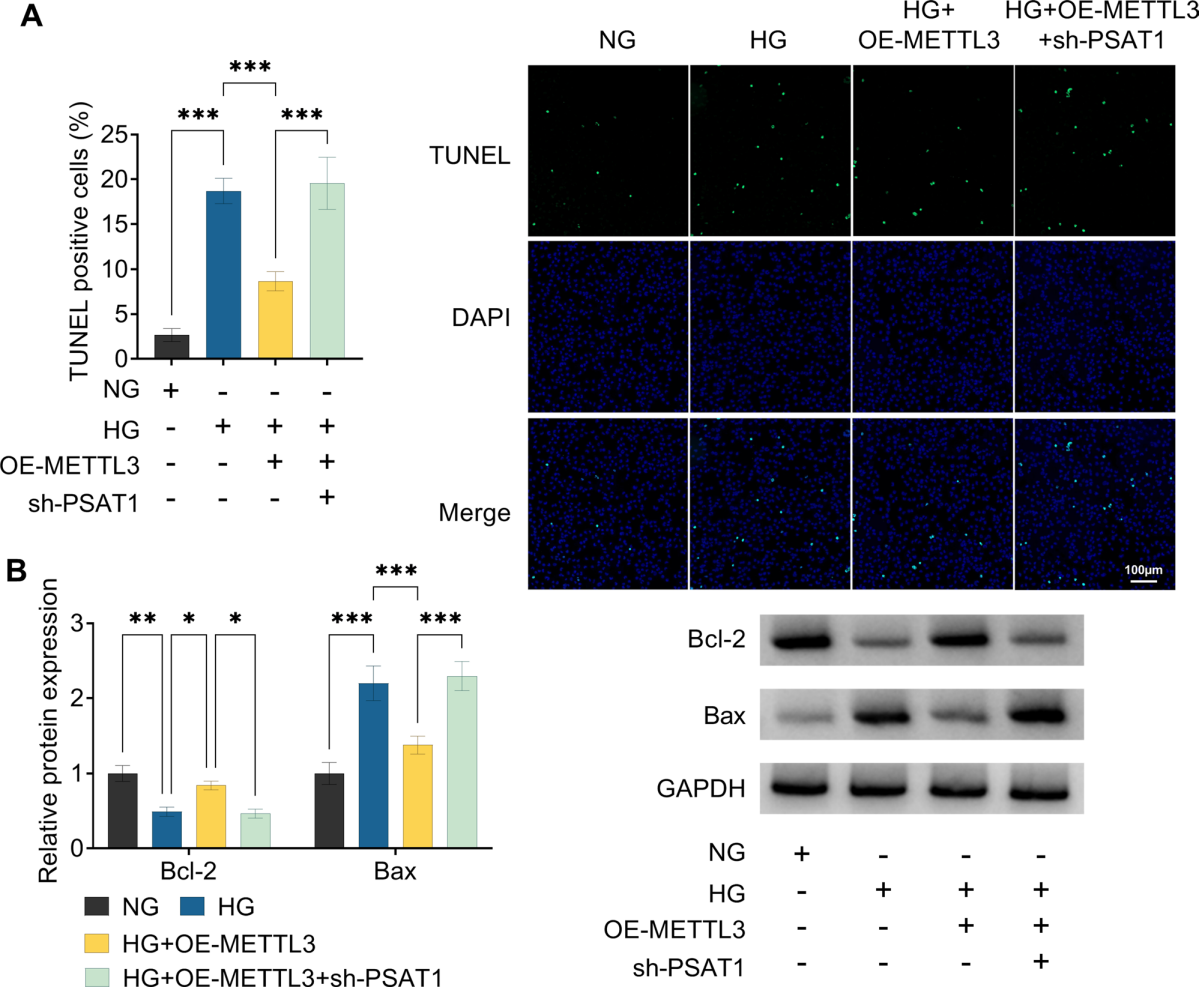
METTL3 relieved HG-induced ARPE-19 cell apoptosis via regulating PSAT1. ARPE-19 cells were treated with NG, HG, HG + sh-PSAT1, or HG + sh-PSAT1 + sh-PSAT1. (**A**) TUNEL analysis of cell apoptosis in treated ARPE-19 cells. (**B**) Western blot assay was used to determine Bcl-2 and Bax protein levels in treated ARPE-19 cells. *P < 0.05, **P < 0.01, ***P < 0.001, *n* = 3

Additionally, to clarify whether PSAT1 might mediate the functional role of METTL3 in HG-treated ARPE-19 cells, we carried out rescue experiments. At first, our data showed that the expression level of PSAT1 was clearly reduced in ARPE-19 cells after sh-PSAT1 introduction (Fig. 5A and B), suggesting that the knockdown efficiency of PSAT1 is successful. After that, CCK-8 assay presented that the co-transfection of sh-PSAT1 might effectively ameliorate METTL3 upregulation-mediated ARPE-19 cell viability increase under HG conditions (Fig. 5C). Meanwhile, PSAT1 deficiency might significantly mitigate the inhibitory effect of METTL3 overexpression on oxidative stress in HG-stimulated ARPE-19 cells, as evidenced by higher ROS and MDA, and lower GSH-Px (Fig. 5D and F). Apart from that, TUNEL assay displayed that OE-METTL3-mediated cell repression was partially weakened by PSAT1 absence in HG-treated ARPE-19 cells (Fig. 6A). In parallel, anti-apoptosis marker Bcl-2 was highly expressed and pro-apoptosis marker Bax was lowly expressed with METTL3 overexpression, which was clearly reversed by PSAT1 downregulation in HG-treated ARPE-19 cells (Fig. 6B). All of these results suggested that PSAT1 knockdown might partly overture the protective effect of METTL3 upregulation on HG-triggered ARPE-19 cell injury.

## Discussion

As a progressive asymptomatic microvascular complication of diabetes, DR can go undetected and unnoticed until irreversible retinal injury and even blindness have occurred [12]. Thus, an effective screening prognostic marker or therapeutic target is necessary for DR pathogenesis. In recent years, progression in the widespread application of high-throughput RNA sequencing and microarrays has allowed transcriptomic researchers to comprehensively verify the function and structure of genes and discover disease-specific identification of biomarkers [11, 30]. Analysis of mRNA molecules and the genes themselves might uncover the functional role of certain genes in human disease.

Herein, bioinformatics methods presented that PSAT1 was of particular interest in this study by detecting microarray data in public databases. Furthermore, our data validated that PSAT1 content was clearly downregulated in DR patients and HG-induced ARPE-19 cells, for the first time. As a transaminase, PSAT1 has been reported to play a vital role in linking metabolic pathways (glycolysis) and amino acid biosynthesis pathways (serine) [33], participating in controlling blood glucose [40]. Meanwhile, the overexpression of PSAT1 might improve insulin signaling and insulin sensitivity, which exerts a critical aspect of maintaining glucose homeostasis [18, 36]. Beyond that, the knockdown of PSAT1 might boost DNA damage and apoptosis [2, 35]. In addition, PSAT1 activation might protect tumor cells from oxidative damage by GSH production [38]. It has been reported that oxidative stress caused by diabetes might play an important role in retinal neovascularization [14]. In the current work, our data exhibited that PSAT1 overexpression might clearly attenuate HG-evoked ARPE-19 cell apoptosis and oxidative stress. These observations provided first-hand evidence that PSAT1 activation might exert a protective effect for HG-induced ARPE-19 cell damage in DR progression.

Additionally, numerous laboratory works have discovered that alteration in m6A modification of RNA might partake in DR development and pathogenesis [13, 25]. Of note, recent literature has suggested that METTL3-meditated m6A modification might govern pericyte dysfunction during diabetes-triggered retinal vascular complications [26]. Moreover, further study displayed that the overexpression of METTL3 might relieve retinal pigment epithelium cell pyroptosis and apoptosis by induced HG through modulating Akt signaling cascade [37]. Consistent with these reports, our data confirmed that METTL3 expression was decreased in DR patients and HG-treated ARPE-19 cells, and its upregulation might weaken HG-induced ARPE-19 cell injury. Regarding the molecular mechanism, METTL3 was a primary RNA m6A methyltransferase that installed the m6A modification and increased the stability of mRNA or non-coding RNA [3, 10]. Herein, our work validated that PSAT1 was a probable downstream target of METTL3-mediated m6A modification in ARPE-19 cells. METTL3 absence might decrease PSAT1 mRNA stability and protein expression in ARPE-19 cells. Then, to further identify whether PSAT1 was the downstream target of METTL3 to regulate HG-mediated ARPE-19 cell damage, rescue assays were performed. As expected, the knockdown of PSAT1 might partly reverse the protective effects of METTL3 upregulation on HG-aroused ARPE-19 cell apoptosis and oxidative stress.

Previous studies have indicated that METTL3 could play different roles in the cell apoptosis of different diseases [9, 10, 32], and we observed an inhibitory effect of METTL3 on ARPE-19 cell apoptosis. One reason for this diversity might be that m6A modifications have diverse decisions on RNA fate, which depends on the m6A reader family YTHDFs [7]. Therefore, in-depth studies are needed to explore whether the reader family YTHDFs could dynamically and spatiotemporally regulate the degradation of PSAT1 mRNA in ARPE-19 cells.

## Conclusion

Taken together, these findings provided compelling evidence that METTL3-mediated PSAT1 mRNA stability increase might alleviate HG-triggered human retinal pigment epithelial cell injury. This research revealed the underlying mechanism of DR pathogenesis, and will provide insight into the clinical discovery of potential agentsfor DR treatment.

## Electronic supplementary material

Below is the link to the electronic supplementary material.

Supplementary Material 1

## Data Availability

No datasets were generated or analysed during the current study.
