# Supplementary material for: PSAT1 is upregulated by METTL3 to attenuate high glucose-induced retinal pigment epithelial cell apoptosis and oxidative stress
Source: Diagn Pathol. 2024 Oct 15;19:138. doi: 10.1186/s13000-024-01556-4 (PMC11476401; doi:10.1186/s13000-024-01556-4)
Supplement: Supplementary file 1 — Supplementary Material 1 [file 13000_2024_1556_MOESM1_ESM.docx]

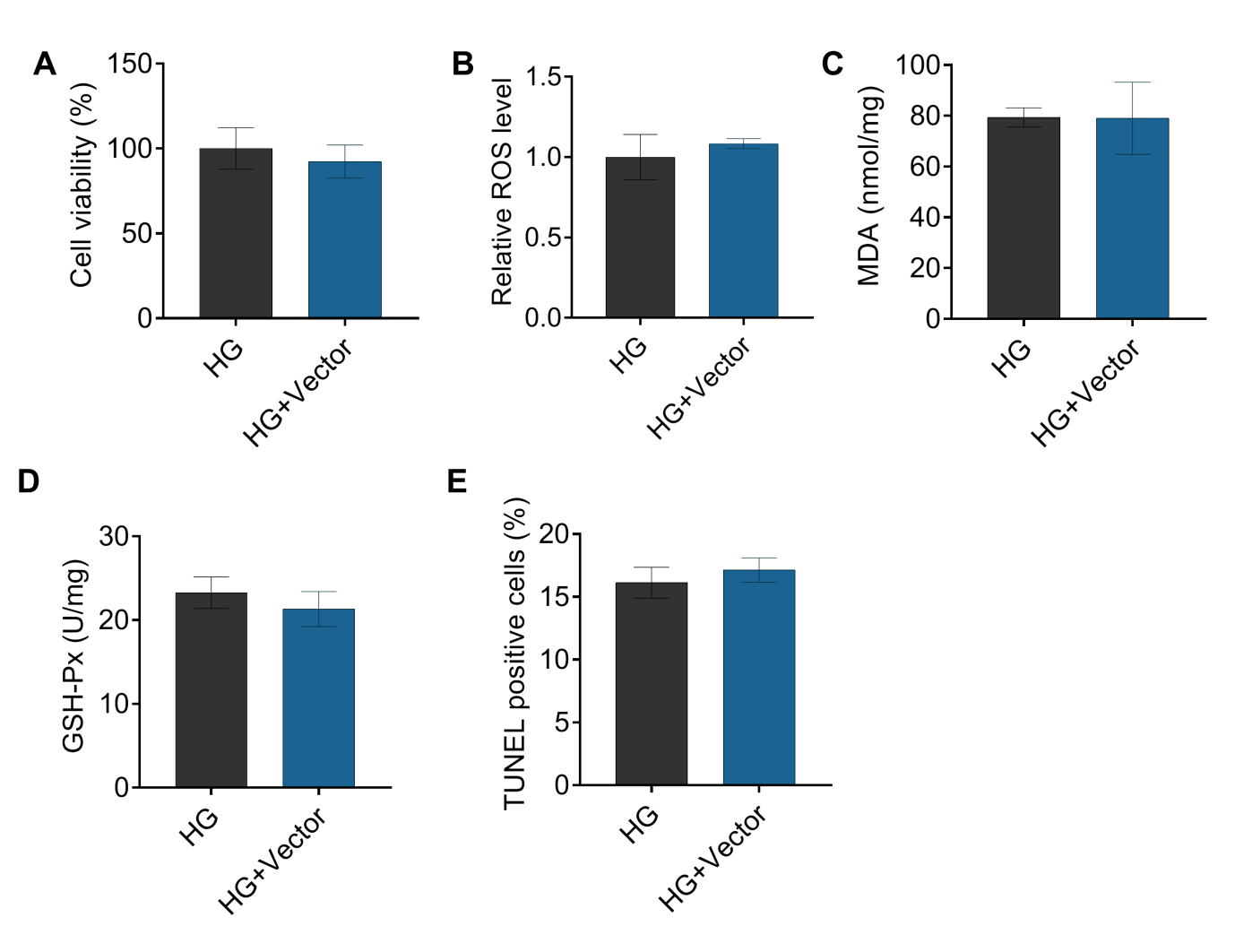


**Figure S1 ARPE-19 cell viability, ROS, MDA, GSH-Px, and apoptosis were detected in the HG group and the HG+Vector group.** ARPE-19 cells were treated with HG or HG+Vector. (A) Cell viability was detected in treated ARPE-19 cells using CCK-8 assay. (B-D) ROS, MDA, and GSH-Px levels were determined using Commercial kits in treated ARPE-19 cells. (E) Cell apoptosis was measured using TUNEL assay in treated ARPE-19 cells. n=3.
